# The First Genetic Map for a Psoraleoid Legume (*Bituminaria bituminosa*) Reveals Highly Conserved Synteny with Phaseoloid Legumes

**DOI:** 10.3390/plants9080973

**Published:** 2020-07-31

**Authors:** Matthew N. Nelson, Jafar S. Jabbari, Rust Turakulov, Aneeta Pradhan, Maria Pazos-Navarro, Jacob S. Stai, Steven B. Cannon, Daniel Real

**Affiliations:** 1 CSIRO Agriculture & Food, Floreat, WA 6014, Australia; 2 The UWA Institute of Agriculture, The University of Western Australia, Perth, WA 6009, Australia; 3 Royal Botanic Gardens Kew, Millennium Seed Bank, Ardingly RH17 6TN, UK; 4 Australian Genome Research Facility, Victorian Comprehensive Cancer Centre, Melbourne, VIC 3000, Australia; jafar.jabbari@agrf.org.au (J.S.J.); rustamzhon.turakulov@anu.edu.au (R.T.); 5 Research School of Biology & Centre for Biodiversity Analysis, 134 Linnaeus Way, Acton, ACT 2601, Australia; 6 School of Biological Sciences, The University of Western Australia, Perth, WA 6009, Australia; aneeta.pradhan@uwa.edu.au; 7 School of Agriculture and Environment, The University of Western Australia, Perth, WA 6009, Australia; maria.pazosnavarro@uwa.edu.au; 8 Interdepartmental Genetics and Genomics Graduate Program, Iowa State University, Ames, IA 50010, USA; jsstai@iastate.edu; 9 USDA-ARS, Corn Insects and Crop Genetics Research Unit, Ames, IA 50011, USA; steven.cannon@ars.usda.gov; 10 Department of Primary Industries and Regional Development, South Perth, WA 6151, Australia; daniel.real@dpird.wa.gov.au

**Keywords:** legume genome evolution, Phaseoleae, Psoraleeae, Ks analysis, linkage mapping, perennial forage legume, tedera

## Abstract

We present the first genetic map of tedera (*Bituminaria bituminosa* (L.) C.H. Stirton), a drought-tolerant forage legume from the Canary Islands with useful pharmaceutical properties. It is also the first genetic map for any species in the tribe Psoraleeae (Fabaceae). The map comprises 2042 genotyping-by-sequencing (GBS) markers distributed across 10 linkage groups, consistent with the haploid chromosome count for this species (*n* = 10). Sequence tags from the markers were used to find homologous matches in the genome sequences of the closely related species in the Phaseoleae tribe: soybean, common bean, and cowpea. No tedera linkage groups align in their entirety to chromosomes in any of these phaseoloid species, but there are long stretches of collinearity that could be used in tedera research for gene discovery purposes using the better-resourced phaseoloid species. Using Ks analysis of a tedera transcriptome against five legume genomes provides an estimated divergence time of 17.4 million years between tedera and soybean. Genomic information and resources developed here will be invaluable for breeding tedera varieties for forage and pharmaceutical purposes.

## 1. Introduction

The Psoraleeae is a tribe of legumes (Fabaceae) distributed across Africa, Europe, Australia and the Americas [1]. It includes economically significant species used in traditional agriculture in North America (*Pediomelum esculentum*, Indian breadroot) [1] and the Canary Islands and Mediterranean region (tedera) [2]. Tedera (*Bituminaria bituminosa* (L.) C.H. Stirton (syn. *Psoralea bituminosa* L.,)) is a perennial forage legume with three botanical varieties (*bituminosa*, *albomarginata*, and *crassiuscula*) native to the Canary Islands [3,4]. Var. *bituminosa* is also widely distributed in the Mediterranean basin and Macaronesia. It is a self-pollinated species [5] with low levels of outcrossing (<8%) when open-pollinated in the presence of insect pollinators [6].

Tedera is a traditional drought-tolerant forage species in the Canary Islands. It has high leaf retention when moisture-stressed, therefore providing valuable livestock feed over the summer and autumn [2,3,4,5,6,7]. This unusual property has attracted much interest in expanding its cultivation in low rainfall areas of Spain and other countries with Mediterranean-like climates such as southern Australia. The Department of Primary Industries and Regional Development (DPIRD) in Western Australia has an active tedera breeding programme to develop commercial cultivars to improve pasture production during the dry seasons [2]. The first commercial cultivar of tedera (Lanza®) was released in 2019 [8].

*Bituminaria bituminosa* var. *bituminosa* is also a source of pharmaceutically active compounds including furanocoumarins (psoralen and angelicin) and pterocarpans in its leaves and other organs [9]. Furanocoumarins are used in the treatment of skin diseases, they encourage antimicrobial activity, and they have anti-HIV effects [10,11,12,13,14,15]. Psoralen can be used in extracorporeal photopheresis for the prevention and treatment of rejection in solid organ transplantation [10,11,12,13,14,15,16]. Pterocarpans have anti-proliferative, estrogenic, hepatic-protective, anti-allergy, anti-inflammatory, apoptotic, and anti-tumour activities [17,18,19].

Tedera is a diploid species (2*n* = 20) [4] with an estimated 2C nuclear DNA content of 0.971–1.040 pg [20]. As a part of the tribe Psoraleeae, tedera is closely related to members of the tribe Phaseoleae including soybean (*Glycine max* (L.) Merr.), common bean (*Phaseolus vulgaris* L.), and cowpea (*Vigna unguiculata* (L.) Walp.) [21]. Tedera breeding and biotechnology could be greatly assisted through accessing the rich genomic information and physical resources generated in these species including high-quality reference genomes [22,23], an RNAseq atlas [24], and characterised mutant stocks [25]. A first step towards accessing soybean information was the development of an expressed gene inventory (transcriptome) for tedera and its comparison with soybean gene models [9]. A more detailed transcriptome has recently become available for tedera through the One Thousand Plant project [26], which can be used to estimate the divergence time between tedera and soybean through Ks analysis [27]. Synteny between the tedera and soybean genomes has so far not been assessed due to the absence of a genetic map for tedera.

Several genotyping methods based on next-generation sequencing have emerged in recent years, which greatly facilitate the development of genetic maps. Restriction site-associated DNA sequencing (RAD-Seq) and genotyping-by-sequencing (GBS) have been used as umbrella terms for techniques which produce a barcoded reduced representation library for sequencing on massively parallel sequencing platforms for high throughput marker discovery and genome-wide genotyping. Genome reduction is achieved by selecting or allowing a subset of restriction fragments to be adapted for sequencing. A range of methods using various sequencing platforms have been described in literature [28,29,30,31,32,33,34,35,36,37,38], and among them, GBS and Double Digest RADseq (ddRAD) offer advantages of simplicity and low cost library prep [30,34]. While GBS workflow is simple and results in libraries with high diversity in initial cycles by using variable length inline barcodes, it suffers from less flexibility to control the number of RAD-tags. In contrast, ddRAD enables tuning tag numbers by choice of restriction enzymes and size-selection window, but it suffers from less diversity in initial sequencing cycles due to use of fixed length barcode. Here, we describe a modified ddRAD method incorporating variable length inline barcodes, which allows considerable control over tag number and produces highly diverse libraries throughout sequencing cycles. 

The objective of this project was to develop a genetic map of the tedera genome, the first for this species and the first for any member of the legume tribe Psoraleeae. We aligned marker sequences from the tedera genetic map with the genome sequences of soybean, common bean, and cowpea to explore genome evolution in psoraleoid and phaseoloid legumes and to provide a resource for molecular marker development for tedera breeding. We also estimate the divergence times between tedera and other legume species using Ks analysis.

## 2. Results

### 2.1. GBS Genotyping

Barcoded GBS libraries were prepared for two tedera parents (1821 and 1281), 185 F2 individuals, and one negative control. Libraries were sequenced in four pools of 48 samples with each parent sequenced three times and all other libraries sequenced once. The first GBS library pool (Pool1) was sequenced on a HiSeq 2000 with v3 chemistry and had lower read output than the other three pools, which were sequenced on HiSeq 2500 v4 chemistry (Table S1). After quality filtering, the average number of reads per sample (excluding parents) was 4.00 M, ranging from 1.61 M to 6.44 M. 

After demultiplexing of sequence tags, the average number of tags was 24,859 (range 20,161 to 36,664), and the average number of unique RAD-tags in the catalogue was 16,333 tags (range 10,060 to 36,072) (Table S2). Catalogue statistics for tag appearance per sample showed that 15,401 tags were shared by 90% of samples (Table S3). 

### 2.2. Linkage Mapping 

**Figure 1:**
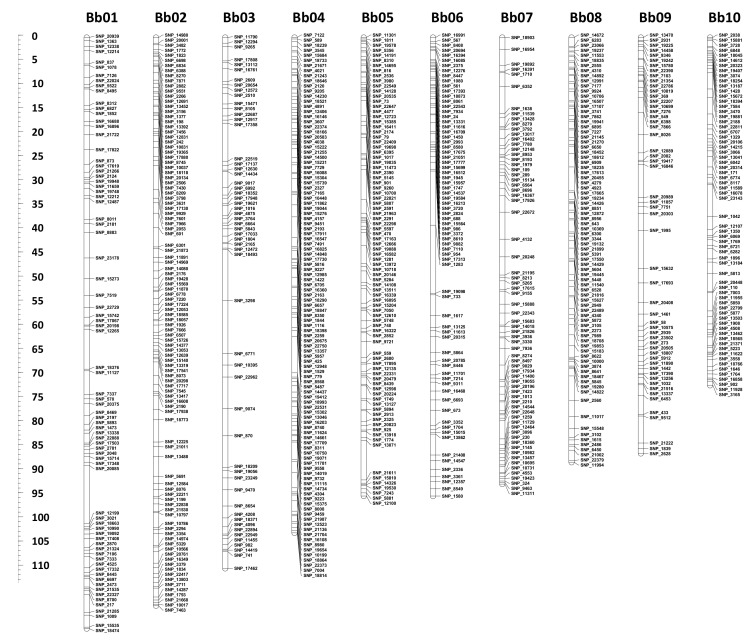
The first genetic map of tedera comprising 2042 markers on 10 linkage groups (Bb01–Bb10). The figure shows the skeleton markers; see Table S4 for full marker details. The centiMorgan scale is provided to the left of the figure.

Preliminary linkage mapping identified seven F2 individuals that exhibited exceptionally high frequencies of double recombinants between adjacent markers; these were removed. The new linkage map of tedera was based on 178 F2 individuals and included 1203 codominant single nucleotide polymorphism (SNP) and 839 dominant presence/absence variant (PAV) markers (2042 markers in total; Table S4). The map comprised ten linkage groups (Bb01–Bb10) spanning 911.3 cM (Table 1, Figure 1). Marker order was deduced using 771 skeleton markers, which were well distributed across the linkage groups with average interval sizes ranging from 0.90 cM (Bb04) to 1.89 cM (Bb03) and maximum interval size from 4.6 cM (Bb02 and Bb08) to 13.1 cM (Bb06) (Table 1). 

### 2.3. Synteny Analysis 

**Figure 2:**
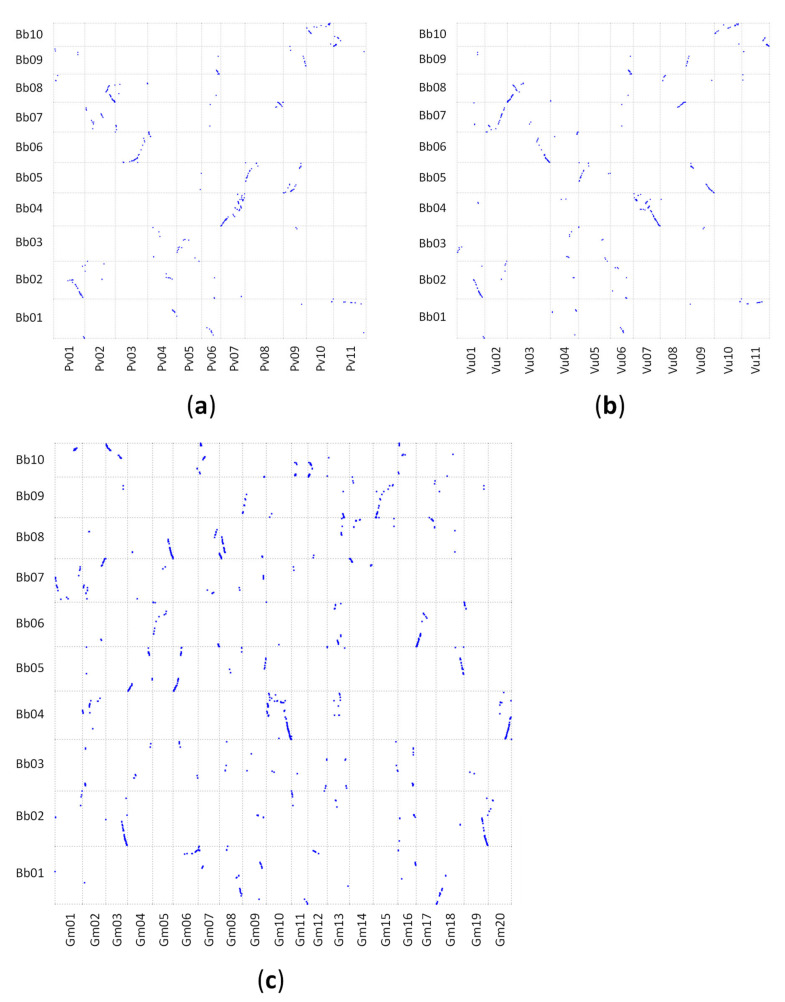
Alignment of the tedera genome (linkage groups Bb01–Bb10) to the phaseoloid genomes of (**a**) common bean (chromosomes Pv01–Pv11); (**b**) cowpea (chromosomes Vu01–Vu11); and (**c**) soybean (chromosomes Gm01–Gm20).

**Figure 3:**
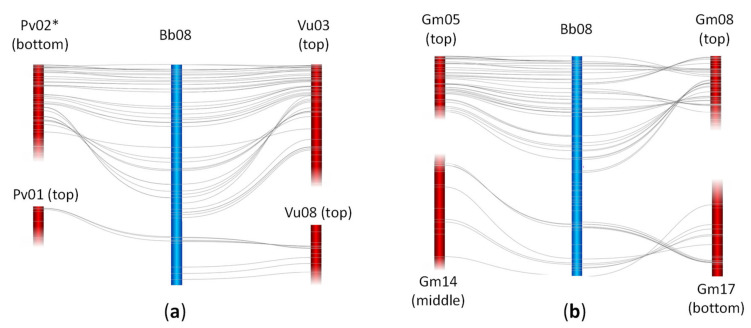
Detailed alignment of tedera linkage group Bb08 with sections of phaseoloid chromosomes of (**a**) common bean Pv02 and Pv01 and cowpea Vu03 and Vu08; (**b**) soybean Gm05, Gm14, Gm08, and Gm17. Asterisk denotes inverted chromosome orientation. Fading at ends of chromosomes indicates chromosome extends beyond the highlighted section.

A homology search using tBLASTx of 2042 tedera GBS markers sequence tags identified 1494, 572, and 587 significant (P < 1e−5) matches in the soybean, common bean, and cowpea reference genomes, respectively (Tables S5–S7). When the genomes were aligned based on these matches, there was conserved synteny between tedera linkage groups and the other legume genomes (Figure 2a–c). Each syntenic block in tedera aligned with a single block in each of common bean and cowpea genomes, and two copies within the soybean genome, consistent with the known whole genome duplication (WGD) in soybean. While syntenic blocks were well-defined, there was evidence of extensive rearrangements between tedera and the diploid phaseoloid species, with each tedera linkage group matching regions in 1–4 chromosomes in diploid phaseoloid genomes (Figure 2a,b). For example, there were two syntenic blocks in tedera linkage group Bb08 that aligned to common bean Pv01/Pv02, and to cowpea Vu03/Vu08 (Figure 3a,b), indicative of an historic chromosome translocation event(s). The same blocks in soybean were similarly divided, as well as being duplicated (arising from the known WGD in soybean), into Gm05/Gm14 and Gm08/Gm17 (Figure 3b). An inversion event was apparent in the centre of Bb08 relative to Pv02, Vu03 and Gm08, but not Gm05, suggesting that the tedera form was the original, unrearranged version (Figure 3). No chromosomes were completely unrearranged between psoraleoid vs. phaseoloid genomes.

### 2.4. Ks Divergence Time Estimates

**Figure 4:**
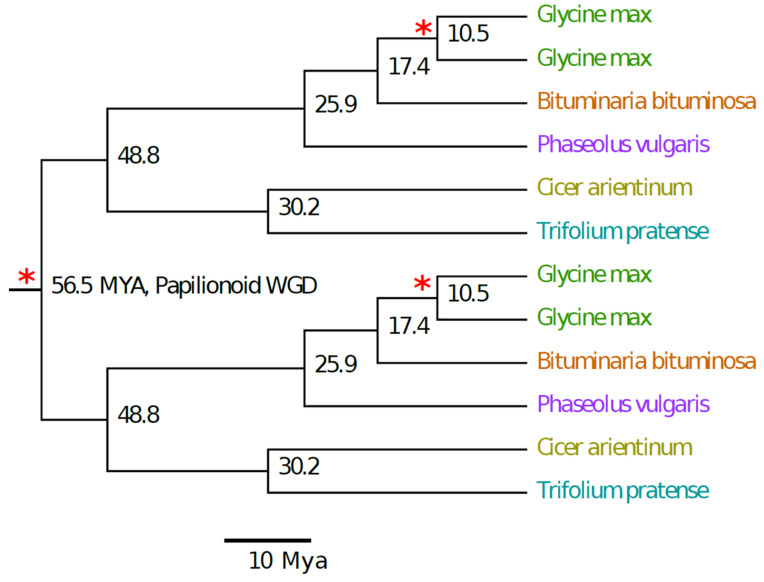
Ultrametric species tree of selected psoraleoid and phaseoloid legumes constructed using a non-negative least squares method, based on synonymous-site changes (Ks values). Ks values for all species pairs were translated to age estimates based on 56.5 Mya for the origin of the papilionoid subfamily and whole genome duplication (WGD). WGDs are indicated by asterisks: one for the papilionoid WGD, and a second for the *Glycine* genus. The species topology was rooted at the midway point of the branch representing the papilionoid whole genome duplication.

A phylogenetic tree was drawn using a distance matrix of Ks peak values for tedera, soybean, common bean, chickpea, and red clover (Figure 4). The branch topology was consistent with phylogenetic expectations [21]. To estimate the temporal depth of the *Bituminaria*-*Glycine* speciation node, we used the date of 56.5 MYA for the divergence time for the deepest node, representing the papilionoid WGD, based on the timing for the origin of the papilionoid clade [39], which is indistinguishable from the timing of the papilionoid WGD [27]. 

The resulting divergence estimates shown in Figure 4 are generally consistent with the corresponding dates reported in Lavin et al. [39]: the *Glycine-Phaseolus* divergence at 19.2 MYA and 25.9 MYA ([39], this study) and the *Cicer-Trifolium* divergence at 28.0 and 30.2 MYA ([39], this study). We infer the *Glycine-Bituminaria* divergence at 17.4 MYA and the *Glycine* WGD at 10.4 MYA. The *Glycine* WGD is close to the estimated dates of 13 MYA and 10.7 MYA reported in analyses of the soybean genome [22] and the soybean and *Phaseolus* genomes together [23], respectively.

## 3. Discussion

Here, we present the first genetic map for tedera and the first for any legume in the Psoraleeae (Figure 1). The GBS method employed was highly effective for generating plentiful, high-quality markers that were well-distributed across the tedera genome. Moreover, a large proportion of the tedera marker sequence tags found significant matches in the genomes of soybean, common bean and cowpea (Tables S5–S7), likely a consequence of using a methylation-sensitive restriction endonuclease (*Pst*I) in the GBS library preparation, which favoured genic regions.

Alignments of the tedera genetic map with the genomes of soybean, common bean and cowpea (Figure 2; Figure 3) indicates that genomic information in these well-studied crops will be relatively straightforward to leverage in tedera. For example, if a simply controlled agronomic or quality trait is mapped to a defined genetic interval in tedera, the equivalent genomic region in genome sequence from other species in the Phaseoleae can be mined for candidate genes with functional annotation consistent with the tedera trait. While the soybean WGD complicates the genome alignment, it shows the smallest number of point mutations with respect to tedera of these phaseoloid species (Figure 4) [21]. Soybean is also the best-resourced and most intensively researched species in the Phaseoleae, because of its agricultural significance [22,24,25]. When gene information is combined with transcriptomic information available for tedera [9,26], candidate homologues can be readily identified through desk-based queries for testing in the lab.

While it was straightforward to align tedera linkage groups to other genomes in the Phaseoleae, no tedera linkage group aligned perfectly to a whole chromosome. A large number of chromosome rearrangements were evident, including chromosome fissions/fusions, inversions and translocations (Figure 2, Figure 3 and Figure 4). These chromosome rearrangements presumably occurred in the time between tedera and soybean lineages diverged, which we estimated to be around 17.4 MYA, just 7.0 MYA before the soybean WGD estimated at 10.4 MYA. Reconstructions of chromosome histories in the legumes [40] indicate that the ancestor of the phaseoloid legumes likely had nine chromosomes, with all tested species (*Cajanus cajan, G. max, Vigna radiata, P. vulgaris*) showing some chromosomal rearrangements relative to the reconstructed ancestor. The same is likely true for tedera. One of the apparently better-conserved chromosomes across all compared species is Bb04, matching *Phaseolus* Pv07, *Vigna* Vu07, and *Glycine* Gm10/Gm11 and Gm02/Gm20. This is also identified in Ren et al. [40] as being unusually well conserved across the papilionoid legumes going back to the common ancestor with *Arachis*, for example.

This map provides a strong foundation for quantitative trait locus (QTL) mapping of genes controlling furanocoumarin content and agronomic traits. The alignment of the tedera genetic map to the soybean genome will provide an access point for exploiting the rich genomic information available in soybean and other legumes, aiding the identification of candidate genes for furanocoumarin biosynthesis in tedera. Candidate genes for furanocoumarin biosynthesis identified based on genetic mapping could be validated by gene expression analysis in low- and high- furanocoumarin producing breeding lines and by association analysis in a large panel of furanocoumarin-characterised tedera accessions. These gene-based markers may accelerate the selection of low furanocoumarin lines for forage varieties and high furanocoumarin lines for pharmaceutical purposes.

Rapid developments in genome sequencing technologies provide a promising avenue for developing a chromosome-level reference genome for tedera, the construction of which would be greatly assisted with the complete genetic map presented here. A tedera genome sequence would be useful for tedera breeding and pharmaceutical industries, and it would serve as a reference more broadly for other species in the Psoraleeae. 

## 4. Materials and Methods 

### 4.1. Experimental Population

The parents used in the experimental crossing were individual plants ‘1218’ and ‘1821’ selected for superior agronomic qualities in Western Australia [2]. Crossing was conducted at DPIRD in an insect-proof glasshouse that was naturally lit. One F1 plant was isolated in a glasshouse and used to produce F2 seeds. DNA was extracted from 206 F2 plants using Illustra Nucleon Phytopure Genomic DNA Extraction Kits (GE Healthcare, Parramatta, Australia). DNA was quantified using Qubit (Thermo Fisher Scientific, Waltham, MA, USA) and DNA quality assessed using standard agarose gel electrophoresis. Further quality control was tested using PicoGreen (Thermo Fisher Scientific, Waltham, MA, USA). In total, 185 F2 samples along with parental controls proceeded to GBS marker genotyping.

### 4.2. GBS Marker Genotyping and Bioinformatics Analysis

We initially prepared non-size selected libraries from eight restriction digests consisting of two rare cutters (*Pst*I or *Eco*RI) with four frequent cutter (*Mse*I, *Msp*I, *Nla*III, or *Hpy*CH4IV) restriction enzymes on a pool of DNA from parents and a progeny and chose *Pst*I-*Nla*III combinations for sample processing. We followed a workflow similar to Peterson et al. [34] in which 200 ng of DNA samples individually were digested with *Pst*I-*Nla*III followed by ligation of variable length barcoded sample specific *Pst*I and a common *Nla*III adapters. Adapter ligated fragments were pooled and size-selected in 260–340 bp window using a BluePippin instrument (Sage Science, Beverly, MA, USA) and amplified by polymerase chain reaction (PCR). Each pool consisted of up to 48 samples including parents and one pool also included a negative control in which no DNA was used (Table S1). Each of four library pools was sequenced in one lane of HiSeq 2000 (Pool1) or HiSeq 2500 (Pools2–4) for 100 cycles. In total, 187 DNA samples were processed.

Sequencing data were analysed using Stacks [41]. We used the ‘process_radtags’ option for demultiplexing samples according to fastq read inline barcodes with zero mismatches and allowed to check for read quality and restriction site presence. Demultiplexed reads of replicated parent samples from all pools were merged into one file for each parent. All demultiplexed reads were trimmed to 90 bp. Stacks of similar reads from each individual was formed using the ustacks option by setting minimum depth of 10 reads and disabling haplotype calls from secondary reads. Catalogue tags were formed using cstacks allowing three mismatches between sample tags. All samples including progenies and both parents were used to build a catalogue of common tags. The genotypes program using default settings was used to extract SNP and presence/absence variant (PAV) calls. A custom Perl script was used to automate all applied Stacks stages, to collect statistics on the intermediate stages for quality control purposes, and to generate a spreadsheet containing the sequence variants and their associated information for all tested samples. The major quality controls metrics used were the numbers of called loci, average read depth for samples and tags, and number of missing tag scores). Outlier samples were excluded from the further consideration and STACKS pipeline rerun with the quality-passed data set. 

### 4.3. Linkage Mapping

Firstly, genotype scores were phased relative to the parental control samples. For SNP markers where one parent sample was heterozygous, phasing was deduced from the homozygous parent score. Where both parents were heterozygous at a SNP, both phases were retained, and the correct phase deduced after linkage mapping relative to adjacent, unambiguously phased markers. With PAV markers, heterozygosity manifested as ‘present’ scores in both parent samples. In those cases, both phases were retained, and the correct phase deduced after mapping these dominant markers relative to adjacent, unambiguously phased markers (both SNP and PAV).

All linkage mapping was conducted with the aid of MultiPoint 3.1 (MultiQTL Ltd, Haifa, Israel), which implements an evolutionary optimisation strategy [42]. A preliminary round of mapping using SNP genotyping data as carried out to identify any F2 individuals that exhibited excessive double crossovers and caused unusual marker order instability consistent with high error rate. Such problematic samples were removed from subsequent analyses. Filtering of PAV markers focused on the frequency of missing value scores as an indicator of marker quality. The lower sequencing read depth of Pool1 samples (Table S1) resulted in elevated frequency of missing values in those samples. Preliminary linkage mapping identified substantially higher incidence of double crossovers in Pool1 samples. As a precautionary measure, all PAV scores for Pool1 samples were discarded. 

Final linkage mapping was performed using filtered SNP and PAV marker scores using the principles outlined in Nelson et al. [43] and Kroc et al. [44]. Given their greater information content, codominant SNP markers were used as priority markers for ordering linkage groups. Redundant markers were binned to improve the efficiency of the analysis. Iterative clustering and ordering procedures were performed at increasing recombination frequencies from rf = 0.10 to 0.20. The stability of marker orders was tested using Jackknife resampling, with destabilising markers removed to leave only the most robust ‘skeleton’ markers for determining marker order. Male and female maps were generated separately and then a consensus map was built. Finally, markers that had been removed from the marker ordering process were assigned (or ‘attached’) to their most likely positions in the consensus map. Interval sizes were expressed as Kosambi centiMorgans.

### 4.4. Comparative Mapping of Tedera and Phaseoloid Genomes

The plots of genetic positions in tedera by genomic positions in soybean, common bean, and cowpea were based on BLAST matches of 90bp tedera GBS marker tag sequences against the indicated target genome sequences. BLAST searches used the tblastx program [45], with -evalue 1e−5 and tabular output format (-outfmt 6). Target genomes were formatted as multifasta sequences, with one chromosome (pseudomolecule) sequence per chromosome, and unanchored scaffolds excluded. For common bean and cowpea, the tblastx results were then filtered to keep the top hit per query (tedera marker) against the respective target genome. For soybean, filtering was modified to allow up to two matches per tedera marker, to allow for the whole-genome duplication in soybean. Specifically, the top match per query-and-target pair was first selected, where query was the marker and the target was the chromosome. Then, the top two hits were selected per query (marker). Empirically, the poorest matches across all three genomes had e-values of 9e−6 and percent identities of 63%, and the average e-values and percent identities were 5e−7 and 85%, respectively. To facilitate plotting the results for the entire genomes (whole linkage map × whole genome), coordinates in the results for each query-target species pair were adjusted to give per-genome (or linkage group) coordinates rather than per-chromosome coordinates. A last filtering step was applied to remove isolated marker matches under these conditions: an ‘isolated’ marker match is one for which only one linkage group-chromosome pairing is observed out of five adjacent markers. For example, in the following pairing (ordered by common bean genomic position), the middle pairing (with tedera linkage group Bb04) would be removed—Bb07×Pv02, Bb07×Pv02, Bb04×Pv02, Bb07×Pv02, Bb07×Pv02. Finally, to generate plots, the gnuplot program was used, following the configuration pattern from the MUMmerplot program [46].

### 4.5. Estimating Genome Divergence Times by Ks Analysis

BLAST protein databases were prepared using the configuration for hash indexing from published genomes of soybean, common bean, chickpea and red clover [22,23,47,48] and transcriptome assembly of tedera [26]. An all-by-all BLAST comparison was performed, with each gene in each species being used as a query against the databases of each species. Only BLAST hits of e-value of 1e−5 or lower were reported from the BLAST package. Pairs were filtered by percent identity, retaining pairs >90%. Pairs were then sorted by query gene and by e-value, and the pairs with the lowest e-value for each query gene were extracted as lists of top matches. The two lists of top-matches for each pair were then combined into total lists for each species pair, such that all genes for both species were used as query to find their top match. Top-match lists for self-self species “pairs” (pairs of a species querying itself) were obtained similarly but discarding instances of a gene matching itself. The Ka/Ks ratio was then calculated for all gene pairs of each species pair using Haibao Tang’s script synonymous_calc.py, available via BSD license at Github [49], which in turn makes use of the software clustalw2 [50], pal2nal [51], and PAML [52]. A histogram of these Ks values was created for all populations of gene pairs. Ks peak values were observed manually and recorded for each species-pair, under the assumption that peak values for self-other species pairs represent speciation distances, and that peak values for self-self species pairs all represent a distance to the most recent shared whole genome duplication.

To infer a phylogenetic tree, a triangular distance matrix of Ks peak values was recorded between all leaves of the undirected graph, including replicates representing gene-population descendants of both halves of the legume and *Glycine* whole genome duplications (WGD) 22. Observed Ks peaks for each species pair and for the *Glycine* WGD were assumed to be representative of both species halves. The tree was calculated using Neighbor-Joining/UPGMA clustering as implemented in neighbor.app of the Phylip bioinformatics package [53].

To calculate branch lengths from our inferred tree, a new triangular distance matrix of Ks peak values was recorded, estimates for the Ks peak value for the papilionoid WGD peak in cross-species comparisons (i.e. a peak representing cross-species paralogs). This distance matrix was used to assign branch lengths onto the previously-affirmed species topology matching phylogenetic expectations, via the ultrametric non-negative least squares method (implemented as ‘nnls.tree’) part of the designTree function in the R package phangorn [40]. The species topology was rooted for the purpose of the analysis at the midway point of the branch representing the papilionoid whole genome duplication.

## Figures and Tables

**Table 1:** Marker and interval details of the new linkage map for tedera.

Linkage Group	Skeleton ^1^	Redundant ^2^	Attached ^3^	Total	Length (cM)	Mean Interval Size ^4^	Max Interval Size ^4^
Bb01	77	62	13	152	113.7	1.50	11.2
Bb02	103	155	6	264	109.3	1.07	4.6
Bb03	55	70	19	144	102.1	1.89	10.2
Bb04	107	181	10	298	95.3	0.90	7.0
Bb05	87	109	12	208	88.2	1.03	7.6
Bb06	72	116	21	209	88.2	1.24	13.1
Bb07	69	117	22	208	85.9	1.26	5.2
Bb08	81	136	8	225	81.7	1.02	4.6
Bb09	54	54	19	127	80	1.51	7.3
Bb10	66	136	5	207	66.9	1.03	6.1
**Total**	**771**	**1136**	**135**	**2042**	**911.3**	**1.20**	**13.1**

^1^ Skeleton markers are high-quality markers, each with a unique position in the genetic map.^2^ Redundant markers have identical positions as their respective skeleton markers.^3^ Attached markers are placed in the most likely intervals between skeleton markers.^4^ Marker distances are calculated between adjacent skeleton markers.

## Supplementary Materials

The following are available online at https://www.mdpi.com/2223-7747/9/8/973/s1, Table S1. Sequencing details for tedera GBS. Table S2. Summary statistics for demultiplexed sample tags. Table S3. Catalogue statistics for sample and tags. Table S4. Graphical genotypes for the first genetic map of tedera (*Bituminaria bituminosa*). Table S5. Alignment of the tedera genetic map (linkage groups Bb01–Bb10) with the genome of soybean (chromosomes Gm01–Gm20). Table S6. Alignment of the tedera genetic map (linkage groups Bb01–Bb10) with the genome of common bean (chromosomes Pv01–Pv11). Table S7. Alignment of the tedera genetic map (linkage groups Bb01–Bb10) with the genome of cowpea (chromosomes Vu01–Vu11).
